# Pemafibrate, a novel selective peroxisome proliferator-activated receptor alpha modulator, improves the pathogenesis in a rodent model of nonalcoholic steatohepatitis

**DOI:** 10.1038/srep42477

**Published:** 2017-02-14

**Authors:** Yasushi Honda, Takaomi Kessoku, Yuji Ogawa, Wataru Tomeno, Kento Imajo, Koji Fujita, Masato Yoneda, Toshiaki Takizawa, Satoru Saito, Yoji Nagashima, Atsushi Nakajima

**Affiliations:** 1 Department of Gastroenterology and Hepatology, Yokohama City University Graduate School of Medicine, Yokohama, Japan; 2 Department of Pharmacology Research, Tokyo New Drug Research Laboratories, Kowa Co. Ltd., Tokyo, Japan; 3 Department of Surgical Pathology, Tokyo Women’s Medical University, Tokyo, Japan

## Abstract

The efficacy of peroxisome proliferator-activated receptor α-agonists (e.g., fibrates) against nonalcoholic fatty liver disease (NAFLD)/nonalcoholic steatohepatitis (NASH) in humans is not known. Pemafibrate is a novel selective peroxisome proliferator-activated receptor α modulator that can maximize the beneficial effects and minimize the adverse effects of fibrates used currently. In a phase-2 study, pemafibrate was shown to improve liver dysfunction in patients with dyslipidaemia. In the present study, we first investigated the effect of pemafibrate on rodent models of NASH. Pemafibrate efficacy was assessed in a diet-induced rodent model of NASH compared with fenofibrate. Pemafibrate and fenofibrate improved obesity, dyslipidaemia, liver dysfunction, and the pathological condition of NASH. Pemafibrate improved insulin resistance and increased energy expenditure significantly. To investigate the effects of pemafibrate, we analysed the gene expressions and protein levels involved in lipid metabolism. We also analysed uncoupling protein 3 (UCP3) expression. Pemafibrate stimulated lipid turnover and upregulated UCP3 expression in the liver. Levels of acyl-CoA oxidase 1 and UCP3 protein were increased by pemafibrate significantly. Pemafibrate can improve the pathogenesis of NASH by modulation of lipid turnover and energy metabolism in the liver. Pemafibrate is a promising therapeutic agent for NAFLD/NASH.

The incidence of nonalcoholic fatty liver disease (NAFLD) is increasing worldwide. NAFLD is an important cause of chronic liver injury^1^. NAFLD ranges from nonalcoholic fatty liver (NAFL) to nonalcoholic steatohepatitis (NASH), cirrhosis, and hepatocellular carcinoma^2^. First-line treatment for NAFLD is lifestyle modification to achieve weight reduction, particularly through diet and exercise^3^. However, weight reduction is very difficult to achieve and maintain and therapeutic agents approved for NAFLD treatment are lacking.

Peroxisome proliferator-activated receptors (PPARs) are members of the nuclear receptor superfamily of ligand-activated transcription factors^4^. PPARs contain three isotypes encoded by PPARα (NR1C1), PPARβ/δ (NR1C2), and PPARγ (NR1C3) genes. Each gene exhibits isoform-specific distribution patterns and functions in tissues^5^. PPARs have important roles in regulation of metabolic homeostasis, inflammation, as well as the growth and differentiation of cells^6^,^7^.

PPARα expression is enriched in hepatocytes. PPARα has key roles in regulation of fatty-acid transport as well as peroxisomal and mitochondrial β-oxidation in the liver^8^,^9^. PPARα knockout mice increase the susceptibility to liver steatosis, inflammation, and hepatocellular carcinoma^10^,^11^,^12^,^13^. Therefore, it has been suggested that PPARα has a protective role against NAFLD pathogenesis. PPARα-agonists have been shown to improve the pathologic condition of NAFLD in various preclinical models^11^,^13^,^14^,^15^,^16^,^17^,^18^,^19^. However, fibrates such as PPARα-agonists are weak and their efficacy is limited (at least in part) by dose-dependent side effects such as elevation of levels of transaminases, homocysteine and creatinine^20^,^21^. Fibrates increase the risk of myopathy, and have been associated with rhabdomyolysis. In addition, the efficacy of fibrates on NASH in humans is not known^22^,^23^,^24^,^25^,^26^.

The next generation of PPARα-agonists is called “selective PPARα modulators” (SPPARMα). They maximize the beneficial effects and minimize the adverse effects of fibrates^27^. Pemafibrate is the first of the SPPARMα to be developed, and has been shown to be safe and efficacious against dyslipidaemia in a phase-2 study^28^. Pemafibrate has not been associated with rhabdomyolysis in Caucasian or Japanese subjects.

Here, we investigated the effect of pemafibrate on rodent models of NASH (methionine choline-deficient (MCD)-fed db/db mice and amylin liver NASH model (AMLN)) in comparison with fenofibrate. AMLN is a diet-induced model of NASH and elicits obesity, insulin resistance, and the three stages of NAFLD (steatosis, steatohepatitis with fibrosis, and cirrhosis) without reliance on genetic mutations, use of toxins, or nutrient deficiency^29^.

## Results

### Effect of pemafibrate on physiologic and biochemical characteristics

We began our studies in MCD-fed db/db mice. Supplementary Table 1 shows the data of MCD-fed db/db mice with or without 4 weeks treatment of pemafibrate. db/db (MCD) mice had increased levels of total cholesterol and aspartate aminotransferase (AST) as well as decreased liver weight and levels of triglycerides. Pemafibrate decreased levels of triglycerides and AST in PEMA-L (db/db) and PEMA-H (db/db) mice. Alanine aminotransferase (ALT) levels tended to decrease upon pemafibrate administration, but not in a significant manner (p = 0.09). Pemafibrate increased liver weight in PEMA-H (db/db) mice. This increase might have been the results of PPARα activation. db/db (MCD) mice increased steatosis, hepatocyte ballooning and the NAFLD Activity Score (NAS). However, they exhibited weak lobular inflammation and showed very little fibrosis.

**Figure 1:**
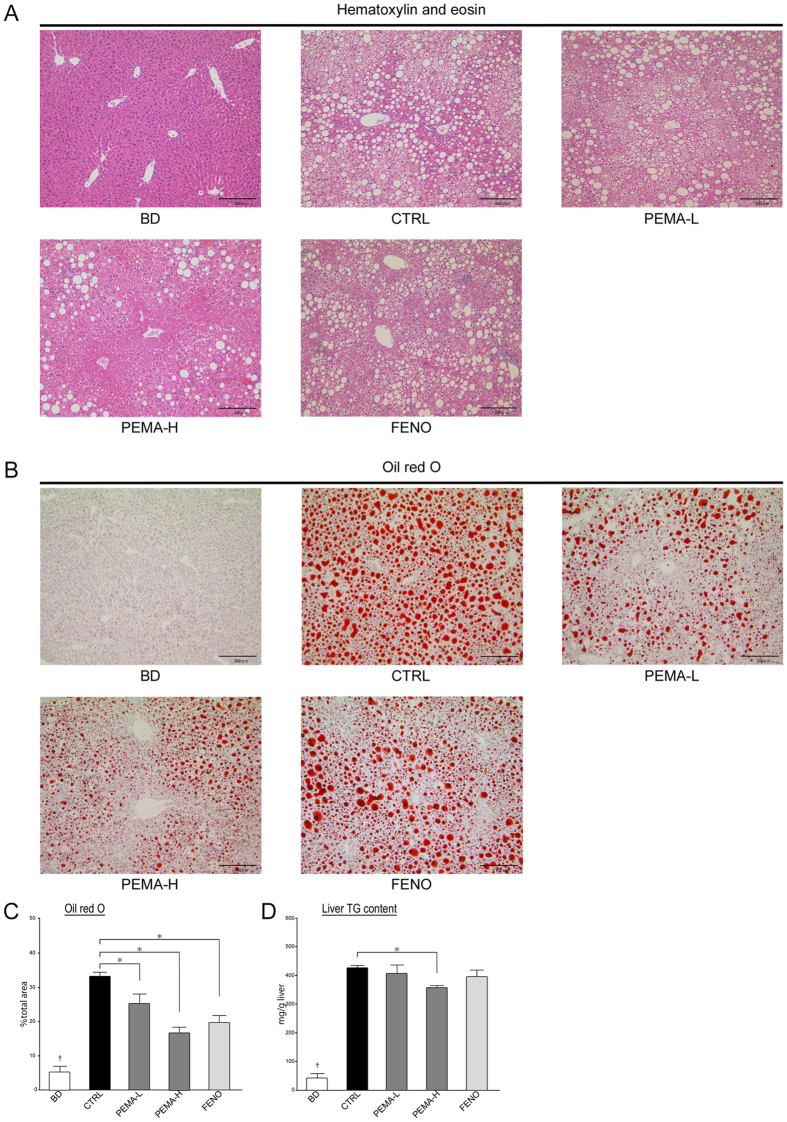
Effect of pemafibrate and fenofibrate on hepatic steatosis. (**A**) Liver sections from BD, CTRL, PEMA-L, PEMA-H, and FENO mice. Haematoxylin and eosin staining, (**B**) oil red O staining. Magnification, 100×. Scale bars: 200 μm. (**C**) Areas of oil red O staining in the liver of BD, CTRL, PEMA-L, PEMA-H, and FENO mice (n = 5). (**D**) Triglycerides content were measured in the livers of BD, CTRL, PEMA-L, PEMA-H, and FENO mice (n = 5). Results are the mean ± SE. Significance was determined using Student’s *t*-test (^†^p < 0.05 *versus* CTRL mice) or Dunnett’s test (*p < 0.05 *versus* CTRL mice).

**Figure 2:**
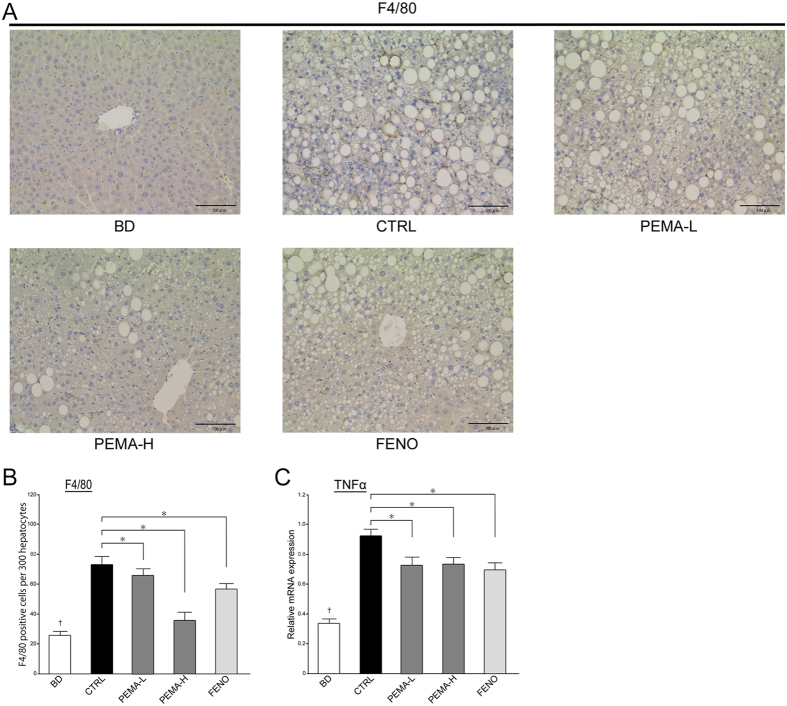
Effect of pemafibrate and fenofibrate on hepatic inflammation. (**A**) Liver sections from BD, CTRL, PEMA-L, PEMA-H, and FENO mice. F4/80 staining. Magnification, 200x. Scale bars: 100 μm. (**B**) The number of macrophages was counted in the livers of BD, CTRL, PEMA-L, PEMA-H, and FENO mice (n = 5). (**C**) Expression of tumour necrosis factor α (TNFα) mRNA in BD, CTRL, PEMA-L, PEMA-H, and FENO mice (n = 5–10). Results are the mean ± SE. Significance was determined using Student’s *t*-test (^†^p < 0.05 *versus* CTRL mice) or Dunnett’s test (*p < 0.05 *versus* CTRL mice).

**Figure 3:**
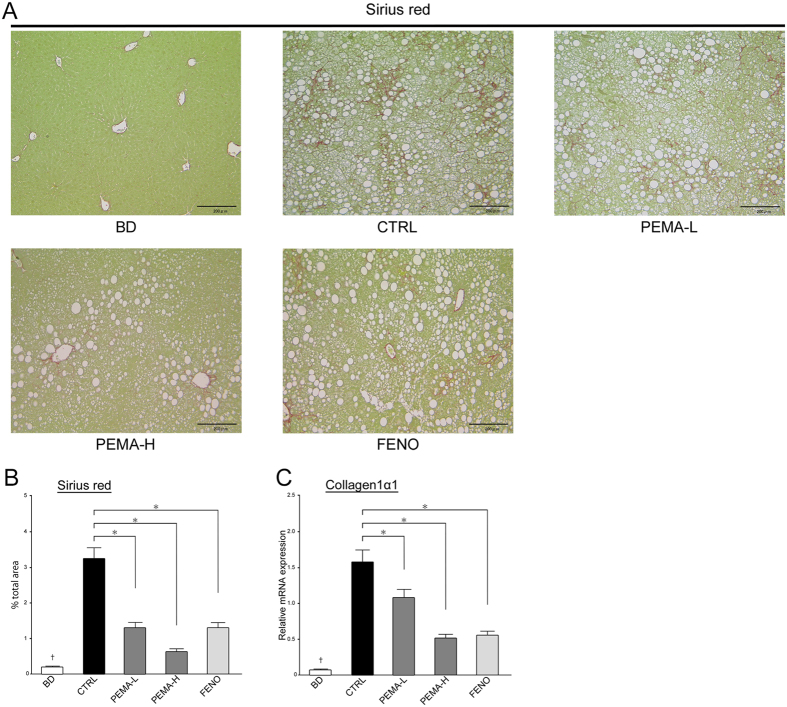
Effect of pemafibrate and fenofibrate on hepatic fibrosis. (**A**) Liver sections from BD, CTRL, PEMA-L, PEMA-H, and FENO mice. Sirius red staining. Magnification, 100x. Scale bars: 200 μm. (**B**) Areas of Sirius red staining in the livers of BD, CTRL, PEMA-L, PEMA-H, and FENO mice were measured (n = 5). (**C**) Expression of collagen 1α1 mRNA in BD, CTRL, PEMA-L, PEMA-H, and FENO mice (n = 5–10). Results are the mean ± SE. Significance was determined using Student’s *t*-test (^†^p < 0.05 *versus* CTRL mice) or Dunnett’s test (*p < 0.05 *versus* CTRL mice).

We continued our investigations using an AMLN because we could not recognize a phenotype of NASH in MCD-fed db/db/mice. Tables 1 and 2 show the characteristics of mice and liver pathology score, respectively. After 20 weeks of feeding of an AMLN diet, CTRL mice exhibited obesity, dyslipidaemia, insulin resistance and liver injury (Table 1). In addition, NASH was clearly evident in CTRL mice, as indicated by steatosis, lobular inflammation, hepatocyte ballooning, and fibrosis. NAS was 7.2 ± 0.2 and fibrosis stage was 2.3 ± 0.2 in CTRL mice (Table 2, Figs 1, 2 and 3).

Food intake was not different among CTRL, PEMA-L, PEMA-H, and FENO mice. Adenosine triphosphate (ATP) content in the liver (p < 0.05) as well as oxygen uptake (VO_2_) (p < 0.01) and production of carbon dioxide (VCO_2_) (p < 0.001) were reduced significantly in CTRL mice. These results showed a reduction of energy expenditure in CTRL mice. There were no differences in liver weight among CTRL, PEMA-L, PEMA-H, or FENO mice. Pemafibrate treatment reduced body weight, as well as the weight of epididymal and subcutaneous adipose tissue. Fenofibrate treatment reduced body weight and the weight of epididymal adipose tissue. PEMA-H and FENO mice showed lower levels of total cholesterol, triglycerides, and free fatty acids. Hyperglycaemia (p < 0.05), hyperinsulinemia (p < 0.05), and insulin resistance (p < 0.01) were improved significantly in PEMA-H mice, but these parameters were unchanged in FENO mice. Treatment with pemafibrate or fenofibrate reduced ALT levels and increased ATP content in the liver. Pemafibrate treatment augmented VO_2_ (p < 0.05) and VCO_2_ (p < 0.05) significantly in PEMA-H mice. However, fenofibrate treatment tended to increase VO_2_ (p = 0.66) and VCO_2_ (p < 0.67), but not significantly. These results suggested that pemafibrate treatment increased energy expenditure markedly.

### Pemafibrate improved the pathogenesis in a rodent model of NASH

Treatment with pemafibrate (p < 0.0001) or fenofibrate (p < 0.05) reduced NAS significantly, but it was lower in PEMA-H mice than in FENO mice. Steatosis grade was significantly lower in PEMA-H (p < 0.001) and FENO (p < 0.01) mice. The area of staining by oil red O was smaller in PEMA-L, PEMA-H, and FENO mice (Fig. 1C). Triglyceride content in the liver decreased significantly in PEMA-H mice (p < 0.05, Fig. 1D). Grade of lobular inflammation tended to decrease in PEMA-L (p = 0.06), PEMA-H (p = 0.11), and FENO (p = 0.28) mice, but these differences were not significant. Fenofibrate treatment did not reduce the grade of hepatocyte ballooning, but pemafibrate treatment reduced it significantly in PEMA-H mice (p < 0.001). The number of infiltrating macrophages and tumour necrosis factor α (TNFα) messenger RNA (mRNA) expression decreased in PEMA-L, PEMA-H, and FENO mice (Fig. 2B,C).

The fibrosis stage decreased significantly in PEMA-H (p < 0.05) and FENO (p < 0.05) mice. The area of Sirius-red staining was smaller and mRNA expression of collagen 1α1 reduced in PEMA-L, PEMA-H, and FENO mice (Fig. 3B,C). These results suggested that pemafibrate and fenofibrate had therapeutic effects on the pathogenesis of NASH.

### Pemafibrate stimulated lipid turnover and upregulated expression of uncoupling protein 3 (UCP 3) in the liver

**Figure 4:**
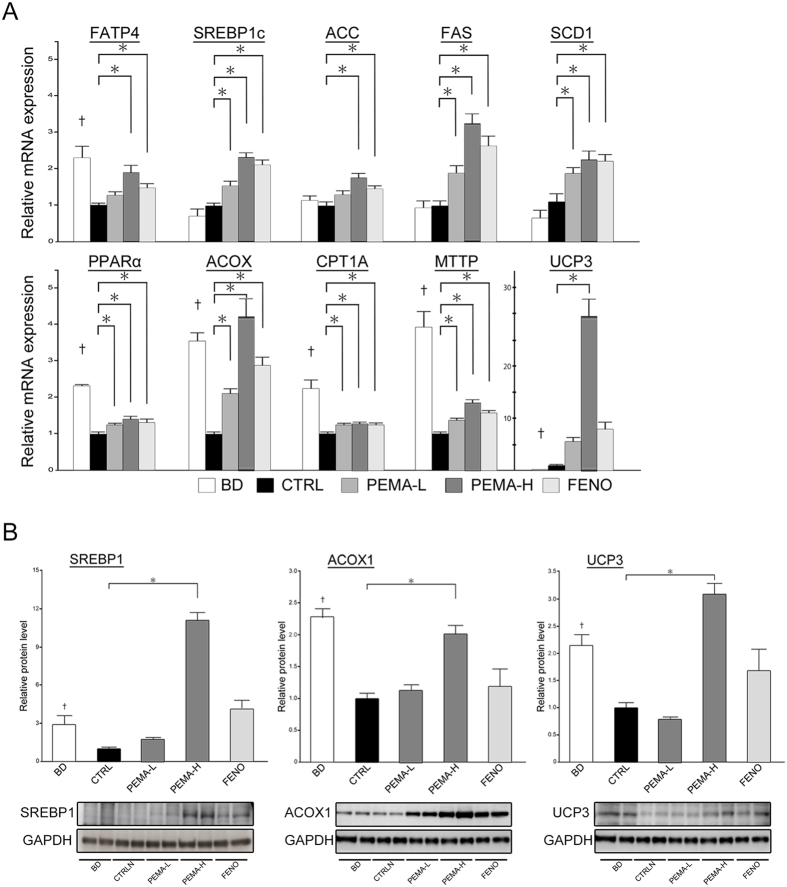
Pemafibrate modulated lipid turnover and upregulated expression of UCP3 in the liver. (**A**) mRNA expressions involved in lipid metabolism (fatty acid transport protein 4 (FATP4), sterol regulatory element-binding protein 1c (SREBP1c), acetyl-CoA carboxylase (ACC), fatty acid synthase (FAS), stearoyl-CoA desaturase 1 (SCD1), peroxisome proliferator activated receptor α (PPARα), acyl-CoA oxidase (ACOX), carnitine palmitoyltransferase 1 A (CPT1A), and microsomal triglyceride transfer protein (MTTP)) and expression of uncoupling protein 3 (UCP3) in BD, CTRL, PEMA-L, PEMA-H, and FENO mice (n = 5–10). (**B**) SREBP1, ACOX1, and UCP3 protein levels in BD, CTRL, PEMA-L, PEMA-H, and FENO mice (n = 5). Results are the mean ± SE. Significance was determined using Student’s *t*-test (^†^p < 0.05 *versus* CTRL mice) or Dunnett’s test (*p < 0.05 *versus* CTRL mice).

We analysed the mRNA and protein expressions of genes involved in fatty-acid transport, lipogenesis, fatty-acid oxidation, and export of very-low-density lipoprotein. We also analysed UCP3 expression in the liver. mRNA expressions of fatty acid transport protein 4 (FATP4) (p < 0.001), PPARα (p < 0.001), acyl-CoA oxidase (ACOX) (p < 0.001), carnitine palmitoyltransferase 1 A (CPT1A) (p < 0.001), and microsomal triglyceride transfer protein (MTTP) (p < 0.001) were significantly lower in CTRL mice (Fig. 4A). mRNA expression of sterol regulatory element-binding protein 1c (SREBP1c), acetyl-CoA carboxylase (ACC), fatty acid synthase (FAS), and stearoyl-CoA desaturase 1 (SCD1) did not differ between BD and CTRL mice. Levels of SREBP1 protein (p < 0.05), ACOX1 protein (p < 0.001) and UCP3 protein (p < 0.001) decreased significantly in CTRL mice (Fig. 4B).

Hepatic fatty-acid transport, fatty-acid oxidation, and export of very-low-density lipoprotein were ameliorated and hepatic lipogenesis were facilitated by treatment with pemafibrate or fenofibrate, as indicated by the increase in mRNA expressions of FATP4, SREBP1c, ACC, FAS, SCD1, ACOX, CPT1A, and MTTP (Fig. 4A). Expression of SREBP1 protein increased in PEMA-H mice (Fig. 4B). Expression of ACOX1 protein increased in PEMA-H mice, but not in FENO mice (Fig. 4B). These results suggested that pemafibrate improved lipid turnover and promoted fatty-acid oxidation notably. Pemafibrate treatment provoked a significant increase in expression the mRNA (p < 0.001) and protein (p < 0.001) of UCP3 in PEMA-H mice (Fig. 4A,B). Fenofibrate treatment tended to increase such expression, but the difference was not significant (p = 0.06 and p = 0.11, respectively).

Fibroblast growth factor 21 (FGF21) is produced mainly by the liver and has insulin-sensitizing activity^30^. K-877 treatment tended to increase serum levels and mRNA expression of FGF21, but not significantly (p = 0.26 and p = 0.91, respectively, Supplementary Fig. 1).

## Discussion

Fibrates (PPARα-agonists) are used to treat dyslipidaemia. It has been suggested that PPARα has a protective role against the pathogenesis of NAFLD. However, the efficacy of fibrates on NAFLD/NASH treatment in humans has not been demonstrated because of study limitations and adverse effects. Pemafibrate is a novel SPPARMα designed to have highly selective and tissue-specific activity without the unwanted side effects of fibrates used currently, and has been developed for dyslipidaemia treatment. Pemafibrate is a more potent PPARα-agonist than fenofibrate (effective concentration inducing 50% response = 1 *vs*. 14000–22400 nM for fenofibrate)^27^. In a phase-2 study, pemafibrate reduced plasma concentrations of liver enzymes (ALT and γ-glutamyl transferase) in patients with dyslipidaemia^28^. Therefore, pemafibrate may have therapeutic efficacy against NAFLD/NASH. Here, we first investigated the effect of pemafibrate on rodent models of NASH.

In the present study, MCD-fed db/db mice and AMLN were used. AMLN is a diet-induced model of NASH. AMLN exhibits the three stages of NAFLD (steatosis, steatohepatitis with fibrosis, and cirrhosis) without reliance on genetic mutations, use of toxins, or nutrient deficiency^29^. The MCD-fed db/db mice model decreased body weight and insulin resistance, and is a useful rodent model for non-obese NASH^31^,^32^. However, fibrosis in db/db (MCD) mice was barely seen, so our main investigations were based on the AMLN.

Supplementary Fig. 2 is a schematic diagram illustrating the effects of pemafibrate on NASH in our study. Fat accumulation in the liver results from imbalanced metabolism of lipids^33^. Recently, it was reported that expression of the PPARα gene in the human liver is correlated negatively with NASH severity. In addition, histologic improvement is associated with an increase in PPARα expression^34^. We have reported that reduction in lipid outflow, fatty-acid oxidation and export of very-low-density lipoprotein are key factors in NASH pathogenesis^35^. In our study, expression of PPARα mRNA decreased and lipid turnover was inhibited strongly in CTRL mice. In addition, CTRL mice exhibited the pathologic condition of NASH. Pemafibrate and fenofibrate improved the pathologic condition of NASH, reduced ALT levels, and inhibited expression of pro-inflammatory and pro-fibrotic genes (F4/80, TNFα, collagen 1α1). Treatment with pemafibrate or fenofibrate increased the expression of PPARα and its target genes, ACOX and CPT1A, significantly. This treatment was thought to increase ATP content in the liver by promoting fatty-acid oxidation, and to improve NASH pathogenesis by stimulating lipid turnover. Therefore, pemafibrate could be a therapeutic agent in NAFLD/NASH, as well as fenofibrate.

Obesity and insulin resistance are important risk factors for NAFLD/NASH. Reports have demonstrated that fenofibrate prevents gain in body weight in genetic or diet-induced models of obesity in rodents^36^,^37^. In the present study, CTRL mice exhibited obesity and insulin resistance. In addition, energy expenditure was reduced, as indicated by a decrease of O_2_ consumption and CO_2_ production in CTRL mice. Pemafibrate and fenofibrate prevented weight gain. Furthermore, pemafibrate increased energy expenditure markedly.

UCPs are members of the mitochondrial anion carrier family. The function of UCPs is to separate oxidative phosphorylation from ATP synthesis by increasing the permeability of the inner membrane of a mitochondrion. UCP3 is expressed primarily in skeletal muscle. Lanni *et al*. reported that fenofibrate increases mRNA levels of UCP3 in the liver^38^. The function of the UCP3 gene is incompletely understood. However, it has been suggested that UCP3 has a protective role against obesity and insulin resistance because it contributes to energy metabolism^39^,^40^,^41^,^42^,^43^,^44^,^45^,^46^,^47^,^48^. It has also been reported that UCP3 polymorphisms are associated with NAFLD^49^. Camara *et al*. investigated the roles of UCP3 in the mitochondria of mouse livers^50^. The presence of UCP3 specifically enhanced an increase of mitochondrial respiratory activity in the presence of palmitate. Camara *et al*. suggested that UCP3 expression increased oxidative capacity and enhanced enzymatic machinery for lipid catabolism in mitochondria. We observed that VO_2_ and VCO_2_ were decreased in CTRL mice compared with BD mice. Pemafibrate provoked a significant increase in expressions of the mRNA and protein of UCP3 in PEMA-H mice. In addition, the respiratory parameters of PEMA-H mice increased to those seen for CTRL mice. This effect might be attributed (at least in part) to improvements in oxidative capacity in mitochondria. These effects participated synergistically to improve energy metabolism and improvement of the pathogenesis of NASH.

Pemafibrate improved glucose metabolism significantly. FGF21 can sensitize insulin and is produced mainly by the liver^30^. In a phase-2 study, pemafibrate increased serum levels of FGF21 significantly^28^. In the present study, pemafibrate tended to increase serum levels and mRNA expression of FGF21. An association of insulin resistance between muscle and adipose tissue cannot be excluded, but weight reduction, increase in energy expenditure, and alternation of FGF21 expression by pemafibrate may contribute to improve glucose metabolism.

Dyslipidaemia is a frequent feature of NAFLD. It has been reported that dyslipidaemia is present in 20–80% of NAFLD patients^51^. NAFLD is also associated with cardiovascular disease^52^,^53^. Fibrates improve dyslipidaemia, reduce levels of triglycerides in plasma, increase levels of high-density lipoprotein-cholesterol in plasma, and lower the risk of major cardiovascular events^54^. However, as mentioned above, fibrates are known to increase levels of transaminases, creatinine and homocysteine. In a phase-2 study in patients with dyslipidaemia, pemafibrate improved lipid parameters without increasing levels of creatinine or homocysteine. Moreover, pemafibrate strongly reduced levels of liver enzymes, whereas fenofibrate did not^28^. Based on the results of that human phase-2 study and our animal study, pemafibrate appears to be a promising therapeutic agent for NAFLD, as well as dyslipidaemia and cardiovascular disease associated with NAFLD.

Our study had two main limitations. First, we used a single dose of fenofibrate (50 mg/kg/day) to compare the therapeutic effect of pemafibrate on NASH, and this dose was based on a report by Fatani and colleagues^55^. Second, there was a discrepancy between improvement of liver steatosis and upregulation of expression of lipogenic transcription factors by treatment with pemafibrate or fenofibrate. Upregulated hepatic lipogenesis is thought to contribute to liver steatosis^56^,^57^. However, the genes involved in lipogenesis did not upregulate/downregulate in expression in CTRL mice or in a report by Shindo *et al*. using fatty liver Shionogi mice^58^. SREBP1c nor its downstream genes (with the exception of SCD1)^59^ have been identified as direct PPARα target genes in mice^60^, but SREBP1c, ACC, FAS, and SCD1 were positively regulated by pemafibrate and fenofibrate in the present study as well in other reports^15^,^59^,^61^,^62^. Hence, alterations in expression of these lipogenic genes may result in feedback by increase in lipid outflow.

We demonstrated that pemafibrate improved the pathogenesis of NASH by stimulation of lipid turnover and upregulation of UCP3 expression in the liver. Pemafibrate is expected to have more beneficial effects on NAFLD/NASH treatment because of its efficacy and safety. Hereafter, large prospective studies investigating the effect of pemafibrate on human NAFLD/NASH are needed.

## Materials and Methods

### Drugs and diets

Pemafibrate and fenofibrate were obtained from Kowa Co. Ltd. (Tokyo, Japan). A basal diet (BD) was prepared containing 22% protein, 6% fat, and 47% carbohydrate. MCD (F2MCD; Oriental Yeast Co., Ltd., Tokyo, Japan) were purchased. A diet rich in fat (40% kcal; Primex partially hydrogenated vegetable oil shortening), fructose (22% by weight), and cholesterol (2% by weight) (catalogue number D09100301; Research Diets, New Brunswick, NJ, USA) was purchased. This diet (the AMLN diet) has been shown to induce all pathologic stages of NAFLD for >20 weeks in C57BL/6J mice^29^.

### Animal experiments

The study protocol was in accordance with the *Guidelines for the care and use of laboratory animals* set by Yokohama City University Medical School (Yokohama, Japan) and was approved by the Committee on the Ethics of Animal Experiments of the same institution.

Surgical procedures were carried out after the induction of anaesthesia using sodium pentobarbital. All efforts were made to minimize animal suffering. Mice were fasted for 12 h and fasting blood glucose measured (Glutest Neo Sensor; Sanwa Kagaku Kenkyusho, Aichi, Japan). The experimental protocol is outlined in Supplementary Fig. 3. Nine-week-old db/db mice (BKS.Cg−+Lepr^db^/+Lepr^db^/Jcl, female) were obtained from CLEA Japan (Tokyo, Japan). After a 2-week acclimatization period, mice were divided into four groups: BD (db/db) mice (fed BD and treated with 0.5% aqueous methylcellulose solution (MC); MCD (db/db) mice (fed MCD and treated with 0.5% MC); PEMA-L (db/db) mice (fed MCD and treated with 0.03 mg/kg pemafibrate); PEMA-H (db/db) mice (fed MCD and treated with 0.1 mg/kg pemafibrate). The drug-free solvent or the dosing solution was administered to animals (5 mL/kg body weight, p.o.) once daily (in the morning) for 4 consecutive weeks (Supplementary Fig. 3A). Six-week-old male C57BL/6J mice were obtained from CLEA Japan. After a 2-week acclimatization period, mice groups were fed according to different regimens. BD mice were fed a BD for 20 weeks. CTRL mice were fed D09100301 for 20 weeks. PEMA-L and PEMA-H mice were fed D09100301 for 12 weeks followed by D09100301 with 0.4 mg and 1.3 mg pemafibrate/kg of the diet for 8 weeks, which corresponded to 0.03 mg/kg/day and 0.1 mg/kg/day, respectively. FENO mice were fed D09100301 for 12 weeks followed by D09100301 with 666.7 mg fenofibrate/kg of the diet for 8 weeks, which corresponded to 50 mg/kg/day (Supplementary Fig. 3B). Pemafibrate and fenofibrate were incorporated into the AMLN diet. Animals were housed under conventional conditions with controlled temperature, humidity, and light (12-h light–dark cycle) and provided with food and water.

### Biochemical analyses

Serum levels of total cholesterol, triglycerides, free fatty acids, AST, and ALT were measured by a local laboratory (SRL, Tokyo, Japan). Serum levels of insulin were measured using a mouse insulin ELISA kit (Morinaga Institute of Biological Sciences, Kanagawa, Japan). As an alternative method for assessment of insulin resistance, the homeostasis model assessment of insulin resistance (HOMA-IR) was calculated using the following formula:


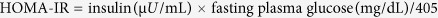


ATP contents in liver tissue were measured by a luciferase assay using an ATP assay kit for animal tissues (TOYO B-Net, Tokyo, Japan). Levels of triglycerides in total lipid extracts of the liver were determined by colorimetric assays (Wako Pure Chemical Industries, Osaka, Japan). Serum levels of FGF21 were measured using a mouse and rat FGF21 ELISA kit (Biovendor, Karasek, Czech Republic).

### Indirect calorimetry

Energy expenditure, VO_2_ and VCO_2_ were measured using a small-animal metabolic measurement system (MK-5000RQ, Muromachi Kikai, Tokyo, Japan). The respiratory quotient was obtained as the ratio of VCO_2_ to VO_2_.

### Histologic and immunohistochemical analyses

Paraffin-embedded sections were stained with haematoxylin and eosin, or Sirius red. The NAS and fibrosis stage were scored by Y.N. in a blinded manner according to the method of Kleiner *et al*. (Supplementary Table 1)^63^. For lipid staining, frozen sections were stained with oil red O and counterstained with haematoxylin. Immunohistochemistry for macrophages was based on F4/80 staining. Immunohistochemistry was carried out on cryostat liver sections (thickness, 7 μm). Sections were incubated with primary antibodies and stained with Alexa Fluor^®^-conjugated secondary antibodies (Cell Signaling Technology, Danvers, MA, USA). To quantify the area of staining by oil red O and Sirius red, images of five random fields from each section were processed with Photoshop Elements v13 (Adobe Systems, San Jose, CA, USA). Each value was expressed as the percentage of the total area of the section. Numbers of F4/80 positive cells were counted and averaged for five random fields of each section.

### RNA isolation and real-time polymerase chain reaction (PCR) analyses

Total RNA was extracted from samples of liver tissue using an RNeasy mini kit (Qiagen, Tokyo, Japan). mRNA of murine TNFα, collagen 1α1, FATP4, SREBP1c, ACC, FAS, SCD1, PPARα, ACOX, CPT1A, MTTP, UCP3, FGF21, and β-actin in liver tissue were determined using a fluorescence-based reverse transcription-PCR and an ABI PRISM 7700 sequence detection system (Life Technologies, Carlsbad, CA, USA).

### Analyses of western blotting

Proteins were incubated with primary antibodies and horseradish-conjugated secondary antibody (Cell Signaling Technology). Primary antibodies were SREBP1, ACOX1 and UCP 3 (Abcam, Cambridge, UK).

### Statistical analyses

Data are the mean ± standard error (SE). Differences between two groups were assessed using Student’s *t*-test or Dunnett’s test. p < 0.05 was considered significant. Statistical analyses were carried out using JMP v11.2.0 (SAS Institute, Cary, NC, USA).

## Additional Information

**How to cite this article**: Honda, Y. *et al*. Pemafibrate, a novel selective peroxisome proliferator-activated receptor alpha modulator, improves the pathogenesis in a rodent model of nonalcoholic steatohepatitis. *Sci. Rep.* **7**, 42477; doi: 10.1038/srep42477 (2017).

**Publisher's note:** Springer Nature remains neutral with regard to jurisdictional claims in published maps and institutional affiliations.

## Supplementary Material

Supplementary Information

## Figures and Tables

**Table 1:** Characteristics of model mice.

Parameter	BD	CTRL	PEMA-L	PEMA-H	FENO
n	5	10	10	10	7
Food intake (g/day)	—	3.28 ± 0.21	3.54 ± 0.25	3.43 ± 0.14	3.02 ± 0.22
Body weight (g)	29.4 ± 0.5^†^	41.3 ± 0.3	39.6 ± 0.4*	36.2 ± 0.3*	38.7 ± 0.4*
Liver weight (g)	1.29 ± 0.05^†^	3.80 ± 0.12	4.00 ± 0.07	3.88 ± 0.09	3.86 ± 0.09
Epididymal adipose tissue (g)	0.32 ± 0.04^†^	1.15 ± 0.04	0.92 ± 0.03*	0.66 ± 0.03*	0.84 ± 0.03*
Subcutaneous adipose tissue (g)	0.19 ± 0.03^†^	0.65 ± 0.03	0.53 ± 0.04*	0.37 ± 0.02*	0.58 ± 0.02
Total cholesterol (mg/dL)	96.8 ± 4.4^†^	283.5 ± 10.2	263.8 ± 6.6	199.5 ± 1.8*	205.0 ± 5.1*
Triglycerides (mg/dL)	61.0 ± 7.5^†^	25.1 ± 1.5	12.6 ± 1.1*	9.1 ± 1.0*	8.6 ± 0.6*
Free fatty acids (uEQ/L)	393.6 ± 42.1^†^	673.2 ± 58.9	494.7 ± 50.2*	332.1 ± 32.4*	274.9 ± 19.5*
Fasting plasma glucose (mg/dL)	95.2 ± 11.4^†^	236.0 ± 9.8	242.6 ± 11.1	194.3 ± 10.9*	223.7 ± 15.9
Insulin (ng/mL)	0.024 ± 0.007^†^	0.096 ± 0.015	0.077 ± 0.002	0.048 ± 0.003*	0.075 ± 0.008
HOMA-IR	0.13 ± 0.04^†^	1.44 ± 0.21	1.13 ± 0.05	0.61 ± 0.07*	0.97 ± 0.16
AST (IU/L)	49.0 ± 6.2^†^	197.6 ± 12.1	210.8 ± 16.0	162.6 ± 14.3	227.1 ± 22.7
ALT (IU/L)	23.2 ± 1.9^†^	285.7 ± 21.0	250.9 ± 16.2	174.3 ± 9.7*	198.7 ± 30.7*
Liver ATP content (μmol/g)^§^	6.59 ± 1.02^†^	4.61 ± 0.34	6.09 ± 0.28*	6.47 ± 0.24*	5.88 ± 0.40*
VO_2_ (mL/min/kg)^§^	53.3 ± 1.7^†^	46.6 ± 0.3	48.1 ± 2.7	52.6 ± 2.2*	48.4 ± 1.6
VCO_2_ (mL/min/kg)^§^	45.0 ± 1.2^†^	36.7 ± 0.8	37.3 ± 1.5	43.5 ± 3.0*	38.6 ± 0.8
RQ^§^	0.85 ± 0.04	0.79 ± 0.01	0.78 ± 0.01	0.83 ± 0.02	0.80 ± 0.03

HOMA-IR, the homeostasis model assessment of insulin resistance; AST, aspartate aminotransferase; ALT, alanine aminotransferase; ATP, Adenosine triphosphate; VO_2_, oxygen uptake; VCO_2_, production of carbon dioxide; RQ, respiratory quotient. Data are the mean ± SE (^§^n = 5). Significance was determined using Student’s *t*-*t*est (^†^p < 0.05 *versus* CTRL mice) or Dunnett’s test (*p < 0.05 *versus* CTRL mice).

**Table 2:** Liver pathology score.

Parameter	BD	CTRL	PEMA-L	PEMA-H	FENO
n	5	10	10	10	7
NAS	0.2 ± 0.2^†^	7.2 ± 0.2	6.2 ± 0.2	3.9 ± 0.5*	5.7 ± 0.4*
Steatosis	0^†^	3	3	1.3 ± 0.2*	2.4 ± 0.2*
Lobular inflammation	0.2 ± 0.2^†^	2.1 ± 0.2	1.2 ± 0.2	1.3 ± 0.4	1.4 ± 0.3
Hepatocyte ballooning	0^†^	2	2	1.3 ± 0.2*	1.9 ± 0.1
Fibrosis	0.4 ± 0.2^†^	2.3 ± 0.2	1.6 ± 0.2	1.4 ± 0.2*	1.3 ± 0.3*

Nonalcoholic fatty liver disease activity score (NAS) and fibrosis stage were scored according to the method described by Kleiner *et al*.^63^, as outlined in (Supplementary Table 2). Significance was determined using Student’s *t*-test (^†^p < 0.05 *versus* CTRL mice) or Dunnett’s test (*p < 0.05 *versus* CTRL mice).
