# Supplementary material for: Pemafibrate, a novel selective peroxisome proliferator-activated receptor alpha modulator, improves the pathogenesis in a rodent model of nonalcoholic steatohepatitis
Source: Sci Rep. 2017 Feb 14;7:42477. doi: 10.1038/srep42477 (PMC5307366; doi:10.1038/srep42477)

Supplementary information

Pemafibrate, a novel selective peroxisome proliferator-activated receptor alpha modulator, improves the pathogenesis in a rodent model of nonalcoholic steatohepatitis

Yasushi Honda[1], Takaomi Kessoku[1], Yuji Ogawa[1], Wataru Tomeno[1], Kento Imajo[1], Koji Fujita[1], Masato Yoneda[1], Toshiaki Takizawa[2], Satoru Saito[1], Yoji Nagashima[3], Atsushi Nakajima[1*]

[1]Department of Gastroenterology and Hepatology, Yokohama City University Graduate School of Medicine, Yokohama, Japan

[2]Department of Pharmacology Research, Tokyo New Drug Research Laboratories, Kowa Co. Ltd., Tokyo, Japan

[3]Department of Surgical Pathology, Tokyo Women's Medical University, Tokyo, Japan

***Corresponding author:**

Atsushi Nakajima, MD, PhD

Department of Gastroenterology and Hepatology, Yokohama City University Graduate School of Medicine, 3-9 Fukuura, Kanazawa-ku, Yokohama 236-0004, Japan

Telephone: +81-45-787-2640; Fax: +81-45-784-3546; E-mail: nakajima-tky@umin.ac.jp

Supplementary Table 1

Characteristics of model mice (methionine choline-deficient (MCD)-fed db/db mice)

Parameter	BD (db/db)	MCD (db/db)	PEMA-L (db/db)	PEMA-H (db/db)
n	8	8	8	8
Body weight (g)	51.4 ± 3.4 †	43.0 ± 1.1	39.3 ± 1.4 *	34.6 ± 2.3 *
Liver weight (g)	2.65 ± 0.37 †	1.25 ± 0.17	1.37 ± 0.13	1.78 ± 0.12 *
Total cholesterol (mg/dL)	114.7 ± 8.7 †	136.7 ± 15.7	147.5 ± 12.8	128.7 ± 15.6
Triglycerides (mg/dL)	80.4 ± 41.8 †	28.9 ± 5.6	21.2 ± 2.5 *	15.9 ± 3.5 *
AST (IU/L)	74.8 ± 12.4 †	87.5 ± 6.5	60.9 ± 4.7 *	48.8 ± 8.1 *
ALT (IU/L)	90.8 ± 16.8	96.7 ± 25.8	61.4 ± 12.3	45.4 ± 13.7
NAS	1.6 ± 1.1 †	4.9 ± 1.0	2.3 ± 1.5	0.9 ± 1.0 *
Steatosis	0.5 ± 0.5 †	2.4 ± 0.7	1.3 ± 0.7	0.5 ± 0.5 *
Lobular inflammation	0.0 ± 0.0	0.6 ± 0.7	0.4 ± 0.5	0.0 ± 0.0 *

Hepatocyte ballooning	1.1 ± 0.6 †	1.9 ± 0.4	0.6 ± 0.9	0.4 ± 0.5

AST, aspartate aminotransferase; ALT, alanine aminotransferase; NAS, nonalcoholic fatty liver disease activity score. NAS was scored according to the method described by Kleiner *et al.*[46], as outlined in Supplementary Table 2. Data are the mean ± SE. Significance was determined using Student's *t*-test (†p < 0.05 *versus* MCD (db/db) mice) or Dunnett's test (*p < 0.05 *versus* MCD (db/db) mice).

Supplementary Table 2

Scoring system and fibrosis stage

NAFLD Activity Score

Item	Definition	Grade
Steatosis	<5%	0
	5–33%	1
	33–66%	2
	>66%	3
Lobular inflammation	None	0
	<2 foci/field	1
	2–4 foci/field	2
	>5 foci/field	3
Hepatocyte ballooning	None	0
	Few ballooned cells	1
	Many ballooned cells	2

Fibrosis stage

Item	Definition	Stage
Fibrosis stage	Isolated perisinusoidal or portal/periportal fibrosis	1
	Perisinusoidal and portal/periportal fibrosis	2
	Bridging fibrosis	3
	Cirrhosis	4

Items, definitions, grades, and stages used in this study

Supplementary Figure 1

Pemafibrate tended to increase serum and mRNA levels of fibroblast growth factor 21 (FGF21)

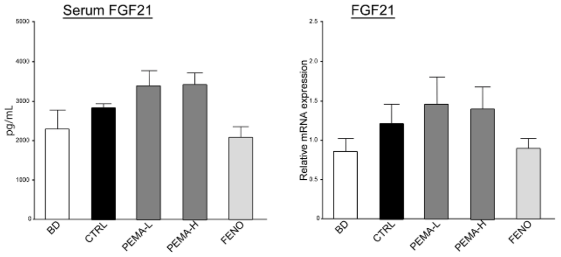

Serum levels and mRNA expression of FGF21 in BD, CTRL, PEMA-L, PEMA-H, and FENO mice (n=5–10). Results are the mean ± SE. Significance was determined using Student's *t*-test (†$p < 0.05$ *versus* CTRL mice) or Dunnett's test (*$p < 0.05$ *versus* CTRL mice).

Supplementary Figure 2

Mechanism detailing the effect of pemafibrate on a rodent model of NASH (schematic)

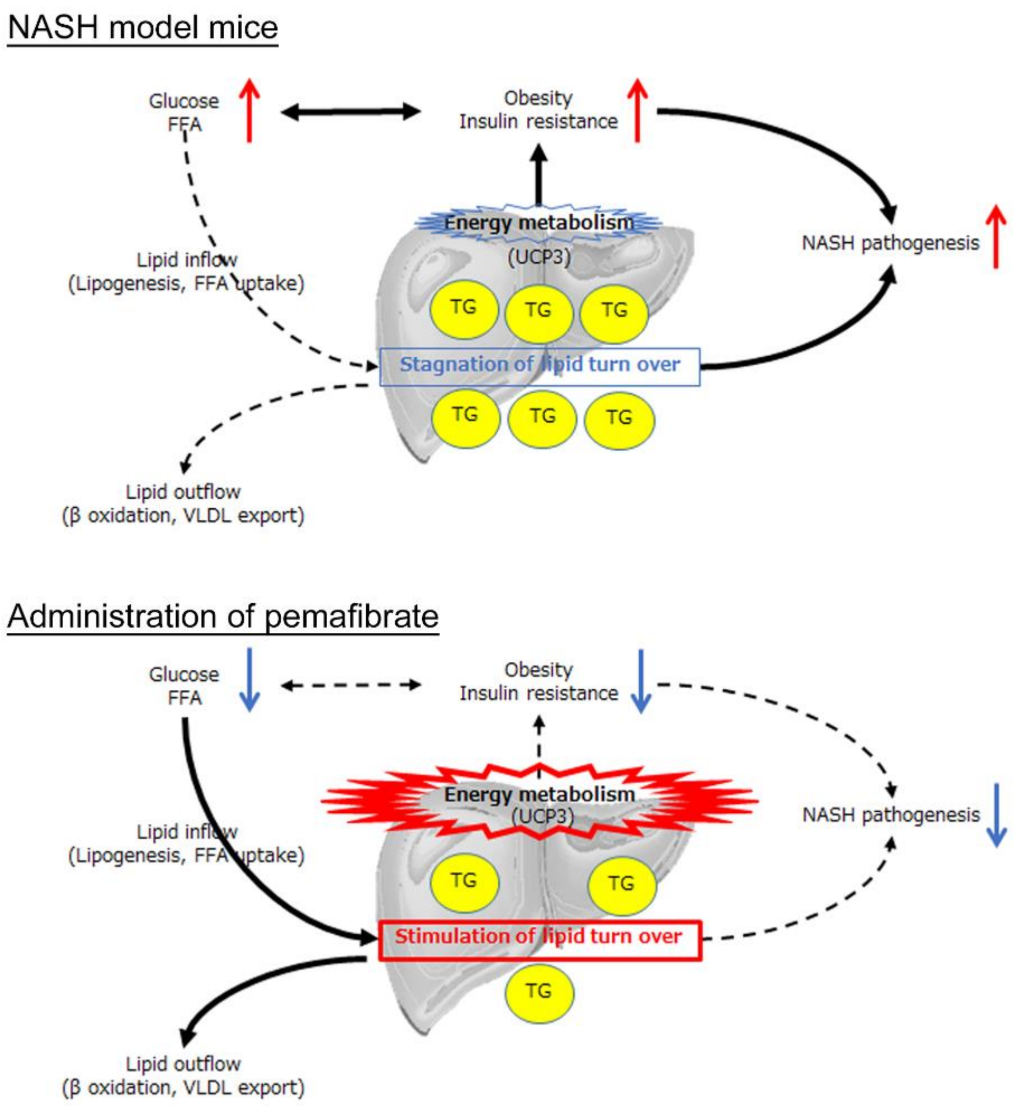

NASH, nonalcoholic steatohepatitis; FFA, free fatty acid; TG, triglyceride; VLDL, very-low-density lipoprotein; UCP3, uncoupling protein 3

Supplementary Figure 3

Experimental protocol

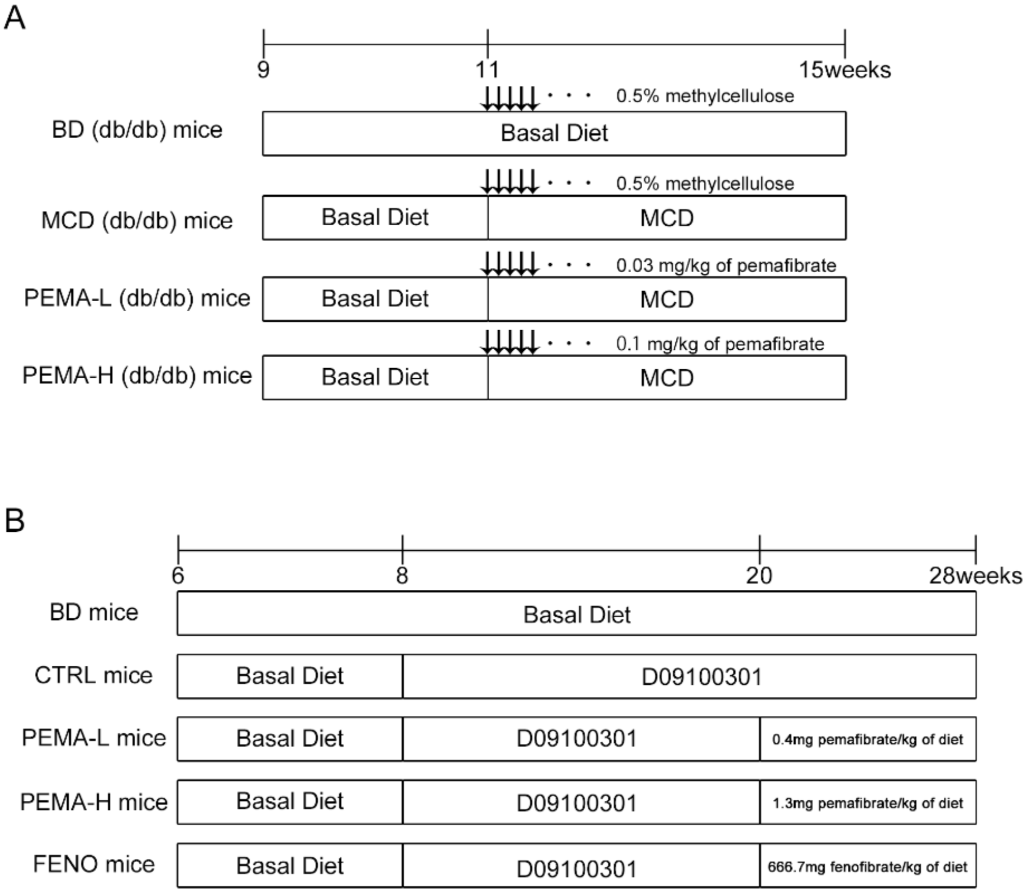

Supplement: Supplementary Information [file srep42477-s1.pdf]
